# The impact of pharmacists’ interventions within the Closed Loop Medication Management process on medication safety: An analysis in a German university hospital

**DOI:** 10.3389/fphar.2022.1030406

**Published:** 2022-11-14

**Authors:** Vivien Berger, Christian Sommer, Peggy Boje, Josef Hollmann, Julia Hummelt, Christina König, Susanne Lezius, Annika van der Linde, Corinna Marhenke, Simone Melzer, Nina Michalowski, Michael Baehr, Claudia Langebrake

**Affiliations:** ^1^ Hospital Pharmacy, University Medical Center Hamburg-Eppendorf, Hamburg, Germany; ^2^ Department of Intensive Care Medicine, University Medical Center Hamburg-Eppendorf, Hamburg, Germany; ^3^ Institute of Medical Biometry and Epidemiology, University Medical Center Hamburg-Eppendorf, Hamburg, Germany; ^4^ Department of Stem Cell Transplantation, University Medical Center Hamburg-Eppendorf, Hamburg, Germany

**Keywords:** pharmacy service (hospital), medication therapy management, medication review, patient safety, closed loop medication management, drug related problems (DRP), pharmacists’ interventions, medication error (ME)

## Abstract

**Background:** Single elements of the Closed Loop Medication Management process (CLMM), including electronic prescribing, involvement of clinical pharmacists (CPs), patient individual logistics and digital administration/documentation, have shown to improve medication safety and patient health outcomes. The impact of the complete CLMM on patient safety, as reflected in pharmacists’ interventions (PIs), is largely unknown.

**Aim:** To evaluate the extent and characterization of routine PIs performed by hospital-wide CPs at a university hospital with an implemented CLMM.

**Methods:** This single-center study included all interventions documented by CPs on five self-chosen working days within 1 month using the validated online-database DokuPIK (Documentation of Pharmacists’ Interventions in the Hospital). Based on different workflows, two groups of CPs were compared. One group operated as a part of the CLMM, the “Closed Loop Clinical Pharmacists” (CL-CPs), while the other group worked less dependent of the CLMM, the “Process Detached Clinical Pharmacists” (PD-CPs). The professional experience and the number of medication reviews were entered in an online survey. Combined pseudonymized datasets were analyzed descriptively after anonymization.

**Results:** A total of 1,329 PIs were documented by nine CPs. Overall CPs intervened in every fifth medication review. The acceptance rate of PIs was 91.9%. The most common reasons were the categories “drugs” (e.g., indication, choice of formulation/drug and documentation/transcription) with 42.7%, followed by “dose” with 29.6%. One-quarter of PIs referred to the therapeutic subgroup “J01 antibacterials for systemic use.” Of the 1,329 underlying PIs, 1,295 were classified as medication errors (MEs) and their vast majority (81.5%) was rated as “error, no harm” (NCC MERP categories B-D). Among PIs performed by CL-CPs (*n* = 1,125), the highest proportion of errors was categorized as B (56.5%), while in the group of PIs from PD-CPs (*n* = 170) errors categorized as C (68.2%) dominated (*p* < 0.001).

**Conclusion:** Our study shows that a structured CLMM enables CPs to perform a high number of medication reviews while detecting and solving MEs at an early stage before they can cause harm to the patient. Based on key quality indicators for medication safety, the complete CLMM provides a suitable framework for the efficient medication management of inpatients.

## Introduction

Drug-related problems (DRPs), defined as events or circumstances in drug therapy that actually or potentially interfere with the intended outcome, lead to preventable negative impact on patients’ safety (Aly, 2015). Apart from an economic burden due to prolonged hospital stays or hospital (re-)admissions, DRPs have negative implications on individual patient outcomes, families and healthcare providers (Moyen et al., 2008; Walsh et al., 2017; Boostani et al., 2019; Elliott et al., 2021). While some patients suffer only temporary harm, others never fully return to their previous health status or even die (James, 2013). Without focusing on specific medical specialties, a recent study found that DRPs were responsible for 45% of hospital admissions (Garin et al., 2021). According to previous studies, about a half or more of DRPs can be prevented (Howard et al., 2007; Leendertse et al., 2008; Zed et al., 2008). Part of the DRPs—especially prescribing errors—can be reduced by the implementation of a computerized physician order entry with clinical decision support system CPOE-CDSS (van den Bemt et al., 2000; Velez-Diaz-Pallares et al., 2018). Further improvement of patient safety can be achieved by implementing of clinical pharmacists (CPs), who can help to solve or prevent DRPs at any stage of the medication process (Fortescue et al., 2003; Bedouch et al., 2009; Zaal et al., 2013; Cornu et al., 2014; Cuvelier et al., 2021). CPs in combination with a Closed Loop Medication Management process (CLMM) are highly recommended by the German Association of Hospital Pharmacists (ADKA) at national level and, furthermore, by the European Association of Hospital Pharmacists (EAHP) (Batista et al., 2020). The CLMM combines electronic prescribing, medication management performed by CPs, patient-orientated pharmaceutical logistics as well as electronic documentation of the administration.

The importance of CPs and their value in the interdisciplinary healthcare team are recognized by various professional societies, especially in the emergency department, intensive care units (ICU), stem cell transplantation (SCT) and as a member of the antimicrobial stewardship program (MacDougall and Polk, 2005; Kumpf et al., 2017; Morgan et al., 2018; Langebrake et al., 2020). While benefits of CPs are becoming more and more visible, clinical pharmacy services are not yet established as a standard in German hospitals. An online survey from 2017 showed that the proportion of hospital pharmacies with integrated clinical pharmacy services was 22% (Schulz et al., 2021). Compared to other European countries, German hospitals are underdeveloped regarding clinical pharmacy services (EAHP, 2019). Additionally, the level of drug supply in Germany is very heterogeneous, with only 8.9% of hospital pharmacies using unit-dose packaging (Schlosser et al., 2020).

In order to strengthen the role of CPs and assess their performance profile, clinical activities need to be documented in a structured way. In Germany, the validated self-reported online documentation and classification system DokuPIK (Documentation of Pharmacists’ Interventions in the Hospital), hosted by the ADKA is available for documentation of DRPs and pharmacists’ interventions (PIs) (Ihbe-Heffinger et al., 2019). In addition to individual in-house evaluations, the ADKA organizes “Intervention Weeks” (IWs) to collect nationwide data on PIs. Through regular analyses and IWs long-term trends of clinical pharmacy services can be examined (Langebrake et al., 2015; Langebrake et al., 2022). Based on a similar approach, PIs in France can be documented on the Act-IP^©^ website (SFPC, 2016). An observational study by Bedouch et al. evaluated PIs documented on this site during a 30-month period to describe the nature of PIs made across all medical specialties and ward types (Bedouch et al., 2015). While nationwide analyses focusing on the relevance of PIs are rare in literature, there are more published single center analyses (Bosma et al., 2008; Zhang et al., 2012; Zaal et al., 2020; Durand et al., 2022). However, international comparison of studies is difficult because of different settings and a lack of standardization in terms of definitions and methods.

Although key quality indicators, like the CLMM with hospital-wide CPs, have already been successfully established at the University Medical Center Hamburg-Eppendorf (UKE), there is no published data on process-related effects on PIs. For this reason, we aimed to assess daily routine interventions to identify the extent of medication reviews, reasons of PIs and acceptance within this process.

## Materials and methods

### Hospital setting and datasets

As a maximum care hospital, the UKE is comprised of 14 centers and more than 80 interdisciplinary cooperating clinics, polyclinics and institutes. The clinic has a total of 1,700 beds and treats approximately 90,000 inpatients and 400,000 outpatients per year. It is located in a metropolitan region with a wide catchment area. Nine CPs of the UKE hospital pharmacy participated in the third DokuPIK IW. They documented all PIs they performed on 5 self-chosen days during a 1-month period (1st to 30th November 2021). Patient-related data (e.g., age, sex, renal and/or liver dysfunction) were entered into DokuPIK completely anonymously. To characterize PIs, involved drugs were classified according to the World Health Organization Anatomical Therapeutic Chemical (ATC) classification (WHO Collaborating Centre for Drug Statistics Methodology, 2021). DokuPIK offers predefined lists with multiple choices for the categories reason, resulting actions and acceptance of the PIs. There are main categories and subcategories for reasons in DokuPIK, which were validated in a prospective survey-based study (Ihbe-Heffinger et al., 2019). The severity of the medication errors (MEs) was assessed according to NCC MERP taxonomy (NCC MERP, 1998). Through a pseudonymous online survey (SoSciSurvey.de), CPs provided information on their professional experience. Additionally, the average number of medication reviews per week was entered, which were defined as “patient days.” Patients can be counted more than once if their medication has been checked on several days per week. For each participating CP, the individual intervention rate was calculated as the performed PIs divided by the reported patient days. For example, when a CP performs 270 medication reviews (= 2 × 60 + 3 × 50) per week and carried out 81 PIs, the intervention rate is 30/100 patient days. Pseudonymized data from the surveys were combined with the pseudonymized data sets from DokuPIK and anonymized before analysis.

### Closed loop medication process at the University Medical Center Hamburg-Eppendorf

The Safe Medication in Time process is based on the principles of the CLMM. In this process, the first step is the electronic prescription by a physician. In the following, prescriptions are checked by CPs and released for unit-dose production in the hospital pharmacy. The last step is the administration and electronic documentation of the medication by nurses. In the UKE, Soarian Clinicals^®^ (Cerner Health Services Deutschland GmbH, Germany, version 4.5.200) is used as an electronic patient record combined with ID MEDICS^®^ (ID Information und Dokumentation im Gesundheitswesen GmbH & Co. KgaA, Berlin, Germany, version 7.8.39.1916) as a CPOE-CDSS including a comprehensive automatic medication check regarding drug-drug interactions, dose, double prescription, intolerance/allergies as well as direct linkage to the unit-dose production and materials management software. The adult ICU and the SCT units document and prescribe within the program Integrated Care Manager (ICM) (Dräger Medical, Lübeck, Germany, version 13.01), with only rudimentary check of dosages and no other automatic medication checks. They are connected to unit-dose supply *via* an interface, but without daily validation of new prescriptions by the CPs.

### Clinical pharmacy services at the University Medical Center Hamburg-Eppendorf

According to different working areas and underlying supply processes, two groups of CPs can be characterized: As part of the CLMM, CPs validate the medication in the CPOE-CDSS twice a day. This affects 83 wards 6 days per week. The CPs in this process, hereafter called the “Closed Loop Clinical Pharmacists” (CL-CPs), focus primarily on newly prescribed medication. The operation mode is defined by standardized workflow stages that are closely timed and tied to logistical processes. Within this process, the first step of the CL-CPs is to look at the prescribed medication and whether it is self-consistent. Previous diseases and further laboratory data are checked in case of special risk drugs, interactions or dose adjustments. If CL-CPs have further questions or consider further interventions, they consult physicians, nurses and/or the patients. According to Geurts et al., in most cases the medication reviews of the CL-CPs can be classified as level 2 (treatment review) (Geurts et al., 2012). Due to this process, CL-CPs record a high number of patient days. In addition, in the adult ICU and SCT wards (*n* = 15), CPs check the whole medication during regular interdisciplinary ward rounds with varying frequency several times per week. The patient population is mainly critically ill with complex medication and laboratory data. The overall review of medication is independent and detached from the CLMM and the associated logistical processes. In the following, this group is named “Process Detached Clinical Pharmacists” (PD-CPs). Based on face-to-face cooperation between CPs, physicians, nurses and/or the patient, the medication reviews can be considered as level 3 (clinical treatment review). Clinical treatment reviews take more time and result in a lower number of patient days. There is no differentiation between full- and part-time CPs considering the number of patient days in either group.

### Medical specialties

The different medical subspecialties were grouped into six specialties: surgery, internal medicine, hematology/oncology, neurology, adult ICU and pediatrics. Ear, nose and throat medicine, urology and gynecology were assigned to the surgical specialty. Dermatology and obstetrics were classified to internal medicine. Radiology/radiation-therapy and SCT have been included in the hematology/oncology category.

### Definitions

Drug-related problems (DRPs) are events or circumstances in drug therapy that actually or potentially interfere with the intended outcome. A medication error (ME) is a deviation from the optimal medication process that leads or could lead to preventable harm for the patient. MEs can affect any step of the medication process and can be caused by anyone involved, especially physicians, pharmacists or nurses. Pharmacists’ interventions (PIs) are defined as actions, discussions or proactive literature research that resolve or avoid DRPs or contribute to an optimal medication use. In this context, the intention of PIs is to influence the physician’s prescription (Aly, 2015; ADKA, 2022a).

### Data analysis

Data were analyzed anonymously using Excel (Microsoft Corporation, Redmond, United States, version 2016) and SPSS Statistics (IBM, Armonk, United States, version 26). CP characteristics were summarized with median and range. PIs and corresponding classifications were analyzed with absolute and relative (percentage) frequencies. For analysis on ATC level, entries with more than one drug involved (e.g., interactions or double prescriptions) were duplicated for further characterization, as both drugs involved were considered equivalent. Analyses on therapeutic subgroups of drugs were provided by using level 2 of the ATC hierarchical classification (therapeutic subgroup). The acceptance of PIs was calculated based on all PIs except those, where only information was provided to physicians or nurses. In all analyses, data entered as “not known” in DokuPIK were treated as missing values. For group comparisons, the correlations of categorical variables were examined using chi-square (χ^2^) or Fisher’s exact test. Ordinal (ranked) variables such as the NCC MERP score for MEs were analyzed using Mann-Whitney U Test. A level of significance of *α* = 0.05 was defined.

## Results

A total of 1,329 PIs were documented by nine CPs of the UKE within the third DokuPIK IW-2021. Five out of these nine CPs are specialized hospital pharmacists according to national further training regulations. The characteristics of the participating CPs are shown in Table 1.

**TABLE 1:** Characteristics of CPs.

Clinical Pharmacist	*n* = 9
Clinical experience in years [median (range)]	4.5 (0.5–15)
Patient days per week [median (range)]	830 (110–1,100)
Intervention rate [median (range)]	21.1 (10.3–79.1)

Depending on the working area and job equivalent of each CP, the number of patient days varied widely. Among their 5,430 reported patient days, the CL-CP subgroup (*n* = 6) documented 1,125 PIs. In contrast, pharmacists of the PD-CP subgroup (*n* = 3) performed 170 PIs for a total of 710 patient days. According to the collected data, CPs intervened in every fifth medication review, corresponding to an intervention rate of 21.1/100 patient days. Excluding those PIs in which only information was provided to physicians or nurses (*n* = 149; 11.2%), the acceptance rate was 91.9%. Non-acceptance was documented due to benefit-risk assessment in 5.4% of the PIs, while proposal rejection occurred in 0.2% and for 0.8% of PIs the outcome remained unknown. The drug related problem could not be solved in 1.7% of PIs.

### Reasons for pharmacists’ interventions

The largest proportion of DRPs was caused by the category “drugs” with 42.7% followed by “dose” with 29.6% (Table 2). Less frequently used reasons for PIs were the categories “other” with 18.7% and “interactions” with 5.5%. The further reasons “contraindication,” “adverse drug reaction,” and “administration” accounted for a total of 3.5%. The most frequently selected reason for a PI with 18.3% was, “(clear) indication, but no drug prescribed.” Other relevant reasons for PIs included “(inappropriate) administration interval” (9.8%), “(inappropriate) dose” (9.9%), and “(clear) indication not (or no longer) given” (9.6%). Transcription errors (0.1%) were almost non-existent. Physicians’ acceptance rate varied between 0 and 100%, which resulted in an overall acceptance rate of PI 91.9%. Outlier values for acceptance rates could be seen especially in the categories with a low number of documented PIs for example in “administration (duration)” (0.0%) and “drug allergy or medical history not considered” (50.0%).

**TABLE 2:** Reasons for PIs and acceptance rate.

Intervention type	Number	Percent [%]	Acceptance rate^a^ [%]
**Administration (total)**	**10**	**0.8**	**75.0**
Administration (route)	4	0.3	100.0
Request/Query concerning administration/compatibility	4	0.3	-
Administration (duration)	1	0.1	0.0
Incompatibility or incorrect preparation/reconstitution	1	0.1	100.0
**Adverse drug reaction (total)**	**4**	**0.3**	**100.0**
**Contraindication (total)**	**32**	**2.4**	**93.8**
**Dose (total)**	**393**	**29.6**	**93.0**
(Inappropriate) administration interval	130	9.8	93.9
(Inappropriate) dose	132	9.9	92.1
Failure to adjust dose for organ dysfunction	79	6.0	90.8
TDM not performed or neglected	52	3.9	96.2
**Drugs (total)**	**568**	**42.7**	**93.0**
(Clear) indication, but no drug prescribed	243	18.3	92.4
(Clear) indication not (or no longer) given	128	9.6	89.6
Inappropriate (or not most suitable) drug in terms of indication	75	5.6	89.2
Inappropriate (or not most suitable) drug formulation in terms of indication	50	3.8	90.0
Double prescription	36	2.7	100.0
Inappropriate (or not most suitable) drug in terms of costs	20	1.5	95.0
Prescription/Documentation incomplete/incorrect	5	0.4	80.0
Generic/Therapeutic substitution	7	0.5	100.0
Drug allergy or medical history not considered	3	0.2	50.0
Transcription error	1	0.1	0.0
**Interaction (total)**	**73**	**5.5**	**95.7**
**Other (total)**	**249**	**18.7**	**89.3**
Procurement/Costs	98	7.4	100.0
Advisory service/Drug choice	86	6.5	83.1
Advisory service/Drug dose	64	4.8	88.9
Failure to discontinue relevant drugs pre-/perioperatively	1	0.1	100.0
**Total**	**1,329**	**100.0**	**91.9**

^a^ Acceptance rate of PIs refers to *n* = 1,180, excluding PIs where only information was provided to physicians or nurses.

### Drugs and therapeutic subgroups

Altogether, 1,512 drugs were involved in the 1,329 PIs. Out of these, 339 different drugs belonging to 65 level 2 ATC groups (therapeutic subgroup) were documented. As shown in Table 3, the top five therapeutic subgroups accounted for about 50% of all entered PIs. One-quarter of PIs was associated to “J01 antibacterials for systemic use.” Out of this group, ampicillin plus sulbactam (3.4%), vancomycin (3.0%), meropenem (2.1%), piperacillin plus tazobactam (1.8%) and cefuroxime (1.6%) were also among the top ten drugs most frequently involved in PIs (Table 4). The second most common therapeutic subgroup was “B01 antithrombotic agents” at 9.7% with the top one enoxaparin (5.4%), followed by “N02 analgesics” at 7.5% with oxycodone (1.9%). The ATC groups “A02 drugs for acid related disorders” with pantoprazole (1.9%) and “C03 diuretics” each accounted for less than 5% of the PIs. The other top ten drugs were macrogol (2.1%) and levothyroxine (2.2%).

**TABLE 3:** Top five therapeutic subgroups for PIs.

ATC	Therapeutic subgroup	Number PIs	Percent [%]
J01	Antibacterials for systemic use	382	25.3
B01	Antithrombotic agents	147	9.7
N02	Analgesics	114	7.5
A02	Drugs for acid-related disorders disorders	56	3.7
C03	Diuretics	49	3.2
	Other therapeutic subgroups (*n* = 60)	764	50.5
	**Top five therapeutic subgroups**	**748**	**49.5**

**TABLE 4:** Top ten drugs for PIs.

Drug	Number PIs	Percent [%]
Enoxaparin	81	5.4
Ampicillin plus sulbactam	51	3.4
Vancomycin	46	3.0
Levothyroxine	33	2.2
Meropenem	31	2.1
Macrogol	31	2.1
Pantoprazole	29	1.9
Oxycodone	29	1.9
Piperacilline plus tazobactam	27	1.8
Cefuroxime	24	1.6
Other drugs (*n* = 329)	1,130	74.7
**Top ten drugs**	**382**	**25.3**

### Medical specialties

**FIGURE 1:**
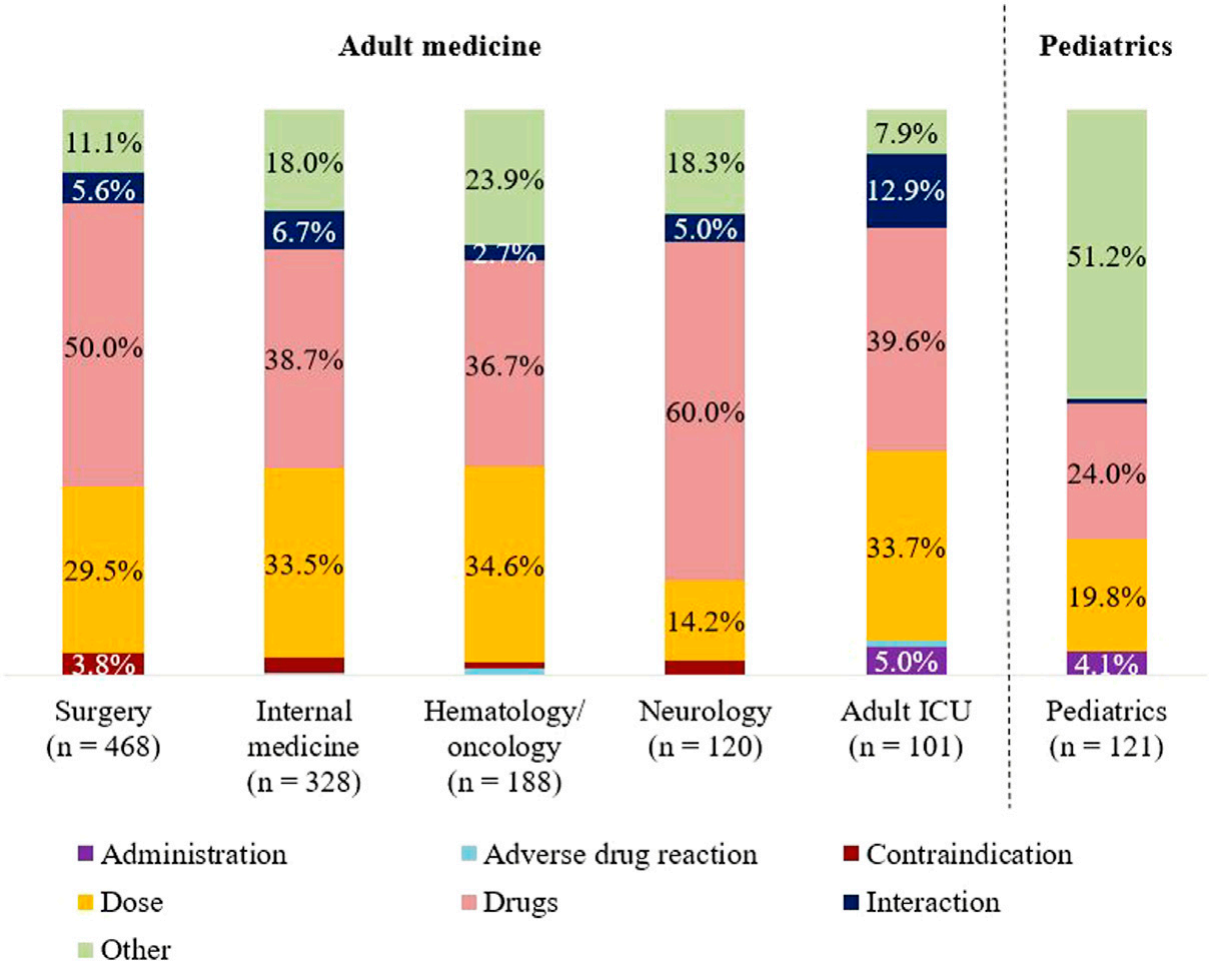
Classification of reasons for PIs: Sorting of specialties (adult vs. pediatrics) by descending number of PIs.

The number of PIs varied depending on the size of department and the patient collective. Frequencies of the reasons for PIs according to the different medical specialties are shown in Figure 1. The largest percentage for the category “drugs” was found in neurology (60%). Dose adjustments were most frequently suggested in hematology/oncology (34.6%) and internal medicine (33.5%). The need for advisory service was most pronounced in pediatrics and was reflected with more than 50% in the category “other.” Compared with adult medicine (summary of the remaining five specialties), the distribution of reasons for PIs was significantly different in pediatrics (*p* < 0.001).

Within each medical specialty, the therapeutic subgroups were similarly distributed. Relevant subgroups involved in PIs were J01, B01, and N02. In four of six specialties, “J01 antibacterials for systemic use” were represented by one-quarter. Exceptions were pediatrics and neurology. In pediatrics, antibiotics accounted for half of the PIs. In contrast, the focus of PIs in neurology was “N02 analgesics” (10.1%).

### Classification of medication errors

Of the total 1,329 PIs, 1,295 (97.4%) were categorized as MEs according to NCC MERP. The vast majority (81.5%) was classified as “error, no harm” (categories B–D), 17.8% as “no error” (category A), and 0.6% as “error, harm or death” (categories E–I). The highest error rating in the IW was category E with a total of eight errors. In these cases, an error occurred that may have contributed to, or resulted in temporary harm to the patient and required initial or prolonged hospitalization. The most common reason for category E was “(clear) indication, but no drug prescribed” (*n* = 5). Further reasons, each provided once, were “(inappropriate) administration interval” or “(inappropriate) dose” and “inappropriate (or not most suitable) drug in terms of indication”. Responsible therapeutic subgroups included “N02 analgesics” (*n* = 4), “A04 antiemetics” (*n* = 3) and “J02 antimycotics for systemic use” (*n* = 1).

**FIGURE 2:**
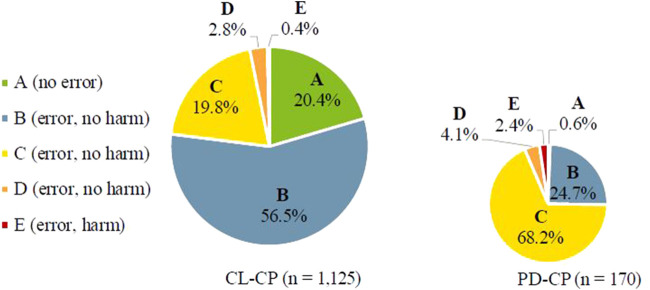
Classification of medication errors according to NCC MERP considering the number of PIs within the two groups of CPs. (**Category A**: Circumstances or events that have the capacity to cause error. **Category B**: An error occurred, but the error did not reach the patient. **Category C**: An error occurred that reached the patient, but did not cause patient harm. **Category D**: An error occurred that reached the patient and required monitoring to confirm that it resulted in no harm to the patient and/or required intervention to preclude harm. **Category E**: An error occurred that may have contributed to or resulted in temporary harm to the patient and required intervention. **Category F**: An error occurred that may have contributed to or resulted in temporary harm to the patient and required initial or prolonged hospitalization. **Category G**: An error occurred that may have contributed to or resulted in permanent patient harm. **Category H**: An error occurred that required intervention necessary to sustain life. **Category I**: An error occurred that may have contributed to or resulted in the patient’s death).

Figure 2 shows the NCC MERP ratings considering the number of PI within the two groups of CPs. In the CL-CP group, the highest proportion was categorized as B errors (56.5%), whereas in the PD-CP group, C errors (68.2%) dominated. There is a highly significant difference in NCC MERP ratings between the subgroups (*p* < 0.001) with a more than threefold higher proportion of C errors in PD-CP group.

## Discussion

In this monocentric analysis, we reported daily routine data on PIs from a university hospital in Germany, where the CLMM and CPs have been implemented hospital-wide for more than a decade. Despite of a comprehensive, CPOE-CDSS, our analysis demonstrates that a huge amount of relevant PIs performed by CPs is still necessary, predominantly with regard to the indication of the drug. For the most part, CPs identified MEs before reaching the patient, which is possible due to regular validation of medication within the CLMM.

Due to closed loop electronic prescribing, more medication reviews become possible during a shorter period of time (Franklin et al., 2007). Our routine data involved a large number of PIs performed by only a few CPs. Compared to the MEDAP study (Kuo et al., 2013)—a cross-sectional observational study, where 62 clinical pharmacists reported 924 PIs during 2 weeks—it is remarkable that in our study 40% more PIs could be documented within half of the time interval (5 days), by significantly less CPs. The higher rate of PIs can be explained by medication reviews within a completely digital CLMM at the UKE.

In another study from Rogers et al. (2016), paper-based prescription charts were checked during a 14-day period by 15 participating organizations in the United Kingdom. They reported 2,782 interventions out of 4,077 medication reviews (68 PI per 100 medication charts). While the number of patient days (*n* = 6,140) is remarkably higher in our data compared to the number of medication charts, the intervention rate reported by Rogers et al. (2016) is more than three times higher. Reasons might be associated to a lack of electronic prescribing in this study. The proportion of MEs due to incomplete prescription, e.g., missing dose or formulation form, was included in the category prescribing errors and not evaluated separately. Therefore, it is difficult to compare results directly.

A first step for physicians in preventing DRPs is to prescribe with the support of a CPOE. While double prescriptions, drug-drug interactions, allergies and overdosing are detected and displayed by CDSS, the alerts must be evaluated by a CP based on the patient’s clinical situation. There are several systematic reviews about the impact of a CPOE to reduce the overall number of DRPs, MEs or adverse drug events (Rothschild, 2004; Rommers et al., 2007; Velez-Diaz-Pallares et al., 2018). Apart from the benefits of CPOE-CDSS, the risk is to miss important reports due to over-alerting. In a prospective follow-up study Zaal et al. (2013) reported that only 1.6% of all alerts from a CPOE-CDSS required a pharmacist intervention. However, new errors such as incorrect drug selection from drop-down menus or wording misinterpretations within CPOE systems can also be generated (Brown et al., 2017).

The high proportion of prescribing errors of 7% found in the MEDAP study might be explained by a missing CPOE-CDSS. In our data, the proportion of errors related to the category “prescription/documentation incomplete/incorrect” is even lower compared to another study from Zaal et al. (2020), where also a CPOE-CDSS was implemented (0.4% vs. 1.2%). Reasons for this could be restrictive default settings of the CPOE at the UKE, where physicians are restricted to the in-house drug list and thus can only prescribe drugs that are actually in stock. Structured datasets make all relevant information available to everyone involved at the point of care. This includes, for example, the type of supply route, information on reimbursement and practical advice on administration. Keeping these up to date requires a high level of data maintenance. Possible causes for an incomplete prescription at the UKE were unstructured datasets in form of notes of physicians in the CPOE-CDSS that did not contain sufficient data on the required medication.

While drug-drug interactions, double prescriptions or transcription errors can be reduced by implementation of a CPOE-CDSS, more complex interventions are expressed by PI regarding questions towards indication and dose adjustment. In our data, reasons for PIs were most frequently classified as drug category (42.7%) and out of these more than one-third was related to PIs with reference to indication. This is comparable to the MEDAP study, where the categories “wrong drug prescribed” and “failure to order needed drug” reached 31.6% of PIs. In contrast, in a French observational study (Bedouch et al., 2015), where 34,522 PIs were documented by 201 CP during a 30-month period, the proportion of PIs referred to reasons of indication was lower with 14.2%. The relatively high proportion at the UKE can be explained by different circumstances. By assigning CPs to specific medical specialties and wards, they can acquire specialist knowledge. Deeply integrated into clinical processes and treatment concepts, more complex interventions can be performed by CPs. In addition to the interprofessional collaboration with physicians and nurses, continuing internal training leads to strengthened knowledge sharing between senior and junior CPs. To reach this level of expertise, it is necessary to perform clinical pharmacy services on a daily basis.

More intensive advisory service to physicians and nurses regarding logistic and administration led to more PI in the category “other” in pediatrics. Particularly in pediatrics, there are more questions about selecting suitable formulations, individual compounding and ordering.

Independent of medical specialty, the therapeutic subgroup “J01 antibacterials for systemic use” were most commonly involved in PIs in our analysis. The high relevance of this group can also be seen in other studies with a proportion of 17%–34% of performed PIs (Rothschild, 2004; Kuo et al., 2013; Bedouch et al., 2015; Salman et al., 2021). Even higher proportion of PI referring to J01 can be seen in pediatrics in our analysis. There are recent studies (Ozdemir et al., 2021; Nasution et al., 2022) addressing the issue of antibiotics in the context of DRP in pediatrics, which underlines the impact of this therapeutic subgroup.

Physicians’ acceptance rates of PIs can be used as a quality indicator to evaluate clinical pharmacy services. The overall acceptance rate of PIs in our data was high with 91.9% and is comparable with those of the MEDAP study (89%). In a retrospective study from Durand et al. (Durand et al., 2022b), they analyzed 2,930 PIs transcribed of CPs over a 6-months period in a French online database (Standardisation et Valorisation des Activités de Pharmacie Clinique, Act-IP^©^). The reported acceptance rate of PIs for all methods of contact was lower with 82.4%, but for PIs submitted verbal or *via* phone call, acceptance rates were higher than 90%. This correlates with the high acceptance rates at the UKE and the primary method of communication for PIs. In most of the cases, CPs submit PIs orally and less frequently *via* prescription software, because this ensures that relevant information reaches the physician. Apart from the communication level, high acceptance rates at the UKE can be explained by well and long established clinical pharmacy services on a daily basis. Comparing with Germany-wide data from the previous IWs, we see increased acceptance rates (IW-2015: 79.9%; IW-2019: 88.4%) over the time, which corresponds with further development of clinical pharmacy services in Germany (Langebrake et al., 2015; Langebrake et al., 2022).

Comparability to other studies is limited because of different settings, methods and classification of PIs and MEs. Regarding NCC MERP classification, most of MEs were rated as “error, no harm” (category B–D) in our data. This correlates with the MEDAP study, where more than 95% did not result in patient harm. The overall high proportion of “error, no harm” in our data can be explained by two aspects. On the one hand, due to the CLMM an increased number of medication reviews become possible by connecting electronic prescribing (including CPOE-CDSS), logistical processes and daily clinical pharmacy services into a time-structured process. This made a high number of patient days possible, especially in the CL-CP subgroup. On the other hand, our analysis gave insights of CLMM towards severity of MEs. Within the CLMM, CL-CPs review new prescriptions twice a day and can react quickly to changes in the course of the day. Consequently, MEs can be detected at an early stage and reach the patient less frequently. This can be seen by a noticeable higher proportion of B errors in the CL-CP group, which is associated with an increased patient safety. A comparison of the results of the national IW in 2019 provides similar results towards differences in number of PI and NCC MERP ratings of UKE (*n* = 5) and non-UKE participants (*n* = 47) (data not published): While the number of documented PIs in both groups were comparable, the severity of MEs differed with two-thirds of MEs within the UKE group classified as B errors, whereas the majority of MEs in the non-UKE group were rated as C errors (42%). Since to the best of our knowledge there is no published data on PIs performed within the CLMM from other hospitals, these results are unique and provide an important basis for implementing the CLMM.

Several limitations of our analysis need to be addressed. First, both groups of CPs were very heterogeneous in terms of clinical experience, type and extent of medication review, different CPOE-CDSS and number of pharmacists. Routine data were only collected over five working days, which might be too short to represent everyday work. Within the IW, only clinical experience of CPs was queried. However, the acceptance of PIs also depends on other factors, such as method of contact (face to face, by telephone, or prescription software) and the status of the physician (postgraduate or resident) (Bedouch et al., 2012; Falcao et al., 2015; Hilgarth, 2019). A correlation between high intervention rates and rising professional experience as found in the nationwide IWs (Langebrake et al., 2022), could not be investigated due to a limited number of CPs in our data. Although the NCC MERP is an internationally accepted method for scoring the severity of MEs, consequences of MEs are measured at the time of the performed PIs and thus do not reflect the relevance of PIs for the patient. For example, during the IW there was a 10-fold overdose of colchicine that was detected by a CP at an early stage before reaching the patient. The alert of the CPOE was ignored by the physician. According to NCC MERP, this ME was classified as a B error, but would have probably been lethal to the patient. This example highlights the need for implementing a classification system into DokuPIK to rate the relevance of PIs. A recently developed classification system is the CLEO (Clinical, Economical and Organizational) tool, which takes into account the clinical impact of PIs from the patient’s point of view (Vo et al., 2021). Therefore, evaluating the relevance of PIs is an important step to further empowering CPs and improving the scalability of clinical pharmacy services.

Through the CLMM, an efficient, safe and cost-effective drug therapy for hospitalized patients can be achieved while encouraging the collaboration of CPs with other healthcare professionals (ADKA, 2022b). The ADKA has set itself the goal of implementing CLMM in all German hospitals within the next 10 years. In accordance with this goal, the role of CPs has been strengthened through legislative changes in the German federal state of Lower Saxony, where CPs became obligatory in 2022.

Necessary digitalization—as a basis of CLMM—is currently being supported in Germany by a law for hospital future, the “Krankenhauszukunftsgesetz” (Bundesgesundheitsministerium, 2020). As recent developments show, there are broad support and relevant reasons for the implementation of CLMM to increase patient safety.

## Conclusion

While digital support using CPOE-CDSS can already optimize the medication process, the combination with CPs may further prevent clinically relevant DRPs. In a setting, where CPs are integrated hospital-wide in a structured CLMM, it is possible for CPs to perform a high number of medication reviews while detecting DRPs at an early stage before they can cause harm to the patient. This real-life data from a long established CLMM highlights the great potential of this process and supports the need to expand its implementation (inter-)nationally. The generalizability of our findings needs to be investigated in future studies, when there is comparable data from further hospital settings and health care systems.

## Data Availability

The original contributions presented in the study are included in the article/supplementary material, further inquiries can be directed to the corresponding author.

## Abbreviations

ADKA, German Association of Hospital Pharmacists; ATC, Anatomical Therapeutic Chemical classification; CL-CP, Closed Loop Clinical Pharmacist; CLEO, Clinical, Economical and Organizational; CLMM, Closed Loop Medication Management process; CP, Clinical Pharmacist; CPOE-CDSS, Computerized Physician Order Entry with Clinical Decision Support System; DokuPIK, Documentation of Pharmacists' Interventions in the Hospital DRP, Drug-related problem; EAHP, European Association of Hospital Pharmacists; ICU, Intensive Care Unit; IW, Intervention Week; ME, Medication Error; NCC MERP, National Coordinating Council for Medication Error Reporting and Prevention; PD-CP, Process Detached Clinical Pharmacist; PI, Pharmacists' intervention; SCT, Stem Cell Transplantation; UKE, University Medical Center Hamburg-Eppendorf.
